# Ultrasound-guided thyroid cartilage plane block for superior laryngeal nerve blockade in awake fiberoptic tracheal intubation: combined cadaveric validation and randomized clinical trial

**DOI:** 10.1097/JS9.0000000000004631

**Published:** 2026-02-02

**Authors:** Tao Shan, Mufeng Gu, Xiao Zhou, Ying Ji, Hongguang Bao, Hongwei Shi, Jie Wei, Qilian Tan, Liu Han, Lihai Chen

**Affiliations:** a Department of Anesthesiology, Perioperative and Pain Medicine, Nanjing First Hospital, Nanjing Medical University, Nanjing, Jiangsu, China; b Department of Human Anatomy, School of Basic Medical Sciences, Nanjing Medical University, Nanjing, Jiangsu, China

**Keywords:** awake tracheal intubation, nerve block, regional anesthesia, superior laryngeal nerve, thyroid cartilage plane, ultrasonography

## Abstract

**Background:**

The thyroid cartilage plane (TCP) block is a novel approach for superior laryngeal nerve (SLN) block in awake tracheal intubation (ATI). We aimed to evaluate the efficacy and safety of TCP block for ATI.

**Materials and methods:**

Our study included a cadaver dissection and a clinical study. Detailed anatomical dissections were performed on two fresh cadavers after a bilateral TCP block with methylene blue. Sixty patients scheduled for ATI were randomized to receive either bilateral TCP block (TCP group) or fiberoptic bronchoscope-assisted topical anesthesia (FIB group) to anesthetize the vocal cords. A 22-gauge needle was advanced targeting the thyroid cartilage laminae, followed by injection of 2.5 ml lidocaine in the TCP group. Lidocaine spray was applied to anesthetize the remainder of the airway. The primary outcome was quality of airway anesthesia grade during intubation (0, excellent; 1, good; 2, fair; 3, poor; 4, very poor), with secondary outcomes including hemodynamic profile, Ramsay sedation score, and incidence of airway hemorrhage and SLN injury.

**Results:**

The methylene blue stained all the internal branches of SLN, with one external branch not stained. In the clinical study, the quality of airway anesthesia was significantly better in the TCP group than in the FIB group (median [IQR], 0 [0–0] vs 1 [0–2], difference [95% CI]: 1 [0–1], *P* < 0.001). Mean arterial pressure and HR were better maintained during intubation in the TCP group (*P* < 0.05). Neither airway hemorrhage nor nerve injury was observed.

**Conclusion:**

Ultrasound-guided TCP block is an effective and safe approach for the SLN blockade, providing an alternative for ATI.

## Introduction

The 2022 American Society of Anesthesiologists Practice Guidelines for management of the difficult airway recommend awake tracheal intubation (ATI) for every anticipated difficult airway^[1]^. This recommendation has significantly increased the clinical application of ATI and, consequently, the demand for anesthesiologists to proficiently master this technique^[2]^.

Adequate airway anesthesia is crucial for ATI. The vocal cords are the most sensitive structures within the airway, and several methods have been used to anesthetize them. The commonest is the fiberoptic bronchoscope(FIB)-guided “spray-as-you-go” techniques^[3–5]^, but patient cooperation, copious secretions, and variable operator experience can limit its reliability^[4,6–8]^.

Bilateral superior laryngeal nerve (SLN) block provides denser anesthesia by abolishing supraglottic sensation and the gag reflex^[8–12]^. However, the classical landmark approach is technically demanding, particularly when anatomical landmarks are obscure, and carries a risk of inadvertent carotid puncture^[13]^. Ultrasound guidance allows for the precise localization of anatomical structures, thereby enhancing the efficacy and safety of SLN block^[14]^. Yet, direct identification of the SLN remains highly variable, with reported success rates ranging from 0% to 100%^[15–20]^. Nerve size, depth of the nerve from skin, and the quality of imaging and needle technology all contribute to this variability^[20–23]^.

Most ultrasound-guided techniques target the 2–3 mm thyrohyoid membrane gap between the hyoid bone and the thyroid cartilage^[19,24,25]^. The SLN and superior laryngeal artery lie within this narrow space, but their small size and anatomical variability make both imaging and injection challenging^[15,20,26]^. Inadvertent intramucosal, intravascular, or intraneural injection remains a real concern, especially for novices.

We previously proposed a novel approach for ultrasound-guided SLN block, termed the thyroid cartilage plane (TCP) block^[27]^. The TCP block instead targets the easily palpable thyroid cartilage lamina and simply injects the local anesthetic along the cartilage surface, eliminating the need to locate the tiny “thyrohyoid membrane gap.” This technique ensures the local anesthetic spreads to the SLN while potentially avoiding unintentional injury to the airway mucosa, nerve, or artery. However, the efficacy and safety of the TCP block have not been fully evaluated.

Therefore, we hypothesized that ultrasound-guided TCP block would be an effective and safe method for SLN blockade. In this study, we aimed to evaluate the efficacy and safety of the ultrasound-guided TCP block in comparison to classic fiberoptic bronchoscope-guided techniques in ATI. Through a combination of anatomical study and clinical trial, we sought to provide a comprehensive assessment of TCP block’s potential as a superior alternative for the management of difficult airways in clinical practice.

## Materials and methods

### Anatomical study

**Figure 1.**
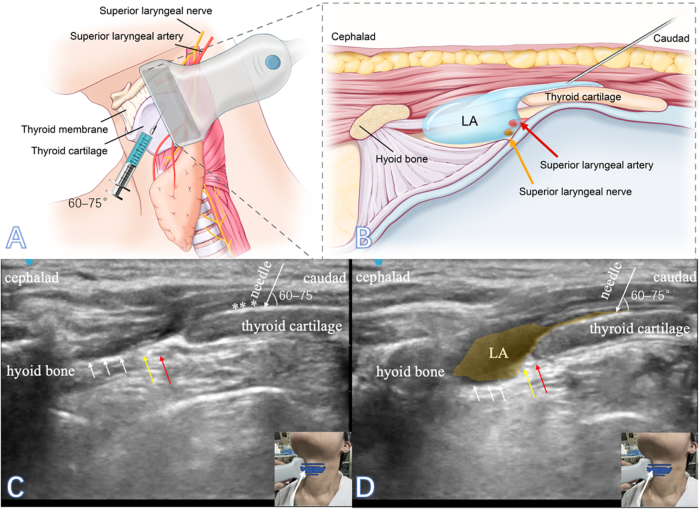
Ultrasound-guided TCP block. A 22-gauge needle was advanced medial to the transducer caudal to cranially at the cranial half of the thyroid cartilage laminae, with out-of-plane technique. (A, B) Anatomical diagram. (C) Pre-injection, with the needle maintaining an angle of 60°–75° to the skin. The white asterisk indicates the saline. (D) Post-injection. LA, local anesthetic. The white long arrow indicates the needle, white short arrow indicated the thyrohyoid membrane, the yellow arrow indicated the superior laryngeal nerve, the red arrow indicated the superior laryngeal artery, and the yellow shadow zone demonstrated spread of LA.

The anatomical study was performed in two fresh cadavers with no history of neck pathology or prior neck surgery (male, 62 years old, 168 cm, 67 kg and female, 67 years old, 159 cm, 63 kg) in accordance with the Ethics Review Board guidelines and after institutional approval. The cadavers were positioned supine with mild neck extension to optimize access to the neck region. A 5–12 MHz linear probe (EDGE II, Sonosite, USA) was placed parasagittally, 30°–45° deviation from the sagittal plane, vertical to the thyroid cartilage, 1.0–1.5 cm lateral to the midline, and aligned with the upper edge of the hyoid bone to locate the laminae of the thyroid cartilage. Using the out-of-plane technique, a 22-gauge needle was advanced medial to the transducer caudal to cranially at the cranial half of the thyroid cartilage laminae with an angle of 60°–75° to the skin until touching the laminae. Correct tip placement in the plane between the cartilage and the overlying muscles was confirmed by injecting 0.5–1 ml saline: real-time elevation of the hyperechoic muscle layer away from the hypoechoic cartilage. Subsequently, a 2.5 ml mixture of 2% lidocaine (2.4 ml) and 1% methylene blue (0.1 ml) was injected within 5 seconds, as shown in Figure 1.

After a 5-minute interval to allow dye diffusion, the cadavers were dissected by the same three anatomists together. The skin, superficial fascia, and platysma muscle were midline incised and reflected laterally. The sternocleidomastoid muscle was identified and retracted laterally to expose the deeper structures. Finally, the SLN and artery were carefully dissected and exposed.

**HIGHLIGHTS**

- This combined study assessed the efficacy of a US-guided thyroid cartilage plane block for superior laryngeal nerve block.
- Anatomical dissection showed consistent dye staining of internal branch of superior laryngeal nerve, and clinical study suggested superior airway anesthesia quality of the thyroid cartilage plane block.
- The thyroid cartilage plane block with the injection site away from the nerve or artery can block the internal branch of the superior laryngeal nerve effectively, providing an alternative method for awake intubation.

### Clinical study

#### Study design and ethics

Our clinical trial was approved by the ethics committee of the hospital on 29 August 2023 (No. KY20230829-10), and registered prior to patient enrollment at ClinicalTrials.gov. Our study was carried out from 15 October 2023 to 27 December 2024. Written informed consent was obtained from all patients before the trial. We adhered to the Transparency in Reporting of Artificial Intelligence 2025 guidance for disclosure of artificial intelligence (AI)-assisted writing^[28]^. No artificial intelligence was employed in any stage of the study design, data analysis, or manuscript preparation.

#### Patients and randomization

We recruited patients aged 18–65 years old, American Society of Anaesthesiologists (ASA) status I–II, who were scheduled for ATI. The inclusion criteria were as follows: (1) limited cervical spine mobility, (2) partial airway obstruction, (3) craniofacial deformities or trauma, (4) micrognathia, (5) mouth opening <3 cm, (6) Mallampati classification III or IV, (7) full stomach. The exclusion criteria were: (1) cardiovascular dysfunction or arterial aneurysms; (2) mental or neurological disorders or concomitant arterial aneurysms; (3) infection at the puncture site; (4) allergy to local anesthetics; (5) continuous use of antiplatelet or anticoagulant medications preoperatively; (6) hoarseness or coughing while drinking water; (7) bronchial asthma; (8) participation in other clinical trials within 3 months; (9) unanticipated transferred to the ntensive care unit (ICU) postoperatively.

We used SPSS 19.0 software to establish a 1:1 allocation ratio for our study participants. The randomization allocation scheme was implemented by an independent nurse who was not involved in the study. This nurse used sealed, numbered opaque envelopes to preserve the allocation results for each participant. The envelopes were numbered in accordance with the sequence of the random number table prior to the commencement of the study, and were sequentially opened when each participant was enrolled. All participants were consecutively assigned to either the thyroid cartilage plane block group (TCP group) or the control group (FIB group).

### Intervention

#### Preparations before airway topical anesthesia

After entering the operating room, ECG, SPO_2,_ and invasive blood pressure were monitored. Sedation was facilitated by a bolus of dexmedetomidine 0.5 μg·kg^−1^ within 5 minutes and remifentanil 0.5 μg·kg^−1^, then continually maintained 0.3 μg·kg^−1^·h^−1^ and 0.1 μg·kg^−1^·min^−1^, respectively, by micropump infusion. The proper anesthesia depth was maintained at 60–80 using the bispectral index throughout the procedure. Additionally, 0.5 mg of Penehyclidine Hydrochloride was injected intravenously once the venous access was established. Throughout the procedure, 100% oxygen 4 L min^−1^ was administered with a nasal cannula throughout the procedure.

### Topical anesthesia

All patients received topical pharyngeal anesthesia using 2.4% lidocaine spray (Xiangxue Pharmaceutical Co. Ltd, China) from the same experienced anesthesiologist. The pharyngeal surface was sprayed twice, with each spray lasting one second and containing approximately 16 mg of lidocaine. This process was repeated after a 5-minute interval. In the fiberoptic bronchoscope (FIB) group, we performed the “spray-as-you-go” technique guided by a fiberoptic bronchoscope as described by Xue *et al*^[6]^ to anesthetize the vocal cord and trachea with 5 ml of 2% lidocaine, respectively. In the TCP group, we performed bilateral TCP block to anesthetize the vocal cord and used “spray-as-you-go” technique with 5 ml of 2% lidocaine to anesthetize the trachea subsequently.

### Ultrasound-guided TCP block

We used a 5–12 MHz linear probe (EDGE II, Sonosite, USA) with a depth typically 15–30 mm, depending on neck habitus, to perform bilateral TCP block. The probe was placed parasagittally, 30°–45° deviation from the sagittal plane, vertical to the thyroid cartilage, 1.0–1.5 cm lateral to the midline, and aligned with the upper edge of the hyoid bone to locate the laminae of the thyroid cartilage. Usually, the thyroid cartilage laminae appeared rough, resembling sandstone on ultrasound imaging. High-echogenicity was often seen at the junction of thyroid cartilage laminae and muscle groups above. After identification of the thyroid cartilage laminae, we advanced a 22-gauge needle medial to the transducer with the bevel of the needle upward, caudal to cranially, at the cranial half of the thyroid cartilage laminae with an angle of 60°–75° to the skin until touching the laminae using the out-of-plane technique. The target depth was 0.5–1 cm from skin to cartilage contact. The correct location of the needle tip in the fascial deep to the muscle groups was confirmed by injecting 0.5–1 ml of saline to view the hypoechoic spread of normal saline between the hyperechoic muscle layer and thyroid cartilage. A total of 2% lidocaine 2.5 ml was administered into the plane between the posterior thyroid cartilage and the anterior muscle groups within 5 seconds after negative aspiration bilaterally. Aspiration was performed on three occasions using a gentle 2-second pull. The procedure was continued only if no blood or air flashback was observed: (1) immediately after needle tip contacts cartilage (before saline test); (2) after saline test (0.5 ml); (3) immediately before local anesthetic injection (2.5 ml) (Fig. 1 and Supplemental Digital Content Video, available at: http://links.lww.com/JS9/G830).

After completing topical anesthesia, we selected a proper disposable reinforced endotracheal tube (TUORen Medical Co, Ltd, China) with internal diameters of 7.5 and 7.0 mm for male and female patients, respectively, and intubated using the fiberoptic bronchoscope. During the whole procedure, respiratory depression was considered if SpO_2_ *<*90%, and the patient was instructed to breathe deeply. All airway topical anesthesia procedures were performed by two experienced anesthesiologists, with 50 cases of this technique completed per year (T.S. and L.C.).

### Outcome measures

The same anesthesiologist, who was blinded to the group allocation, entered the operating room after the completion of airway topical anesthesia and assessed all outcomes. The primary outcome was the quality of airway anesthesia assessed on a 5-point scale^[29,30]^, and graded as follows: 0. excellent, no coughing or gagging in response to intubation; 1, good, mild coughing and/or gagging that did not hinder intubation; 2, fair, moderate coughing and/or gagging that minimally interfered with intubation; 3, poor, severe coughing and/or gagging causing significant intubation difficulty; and 4, very poor, very severe coughing and/or gagging that required additional local anesthetic and/or change in technique.

The secondary outcomes were: (1) tube tolerance score after intubation^[31]^. This score assessed the patients’ tolerance to the endotracheal tube after intubation. The scale ranged from 1 to 3, with the following definitions: 1, cooperation; 2, restlessness and mild resistance; and 3, severe resistance requiring immediate general anesthesia. (2) Time taken to finish the TCP block procedure (defined as the time interval from the placement of the transducer on the skin to the operator’s declaration of completion of injection). (3) Mean arterial pressure (MAP), heart rate (HR), and Ramsay sedation score^[32]^. Satisfactory sedation level was defined as a score of 2–4. These parameters were recorded at different time points, including upon entering the operating room (T_0_), immediately before intubation (T_1_), immediately after endotracheal intubation (T_2_), and 5 minutes after intubation (T_3_). (4) Incidence of sore throat. Sore throat was defined as continuous throat pain and graded 0–1 (0 = none, no sore throat, 1 = continuous throat pain). The occurrence of sore throat was recorded at 1 and 24 h after extubation. (5) Incidence of patient hoarseness. Hoarseness was defined as an acoustic quality differing from the preoperative voice, graded 0–1 (0 = normal, 1 = noticeable by patients or observer, or aphonia) while the patient counted from 1 to 10 and assessed before intubation, 1 and 24 h after extubation. (6) Airway hemorrhage immediately before intubation (observation of bleeding of the laryngeal mucosa by fiberoptic bronchoscope), 1 and 24 h after extubation (observation of bloody secretions via coughing). (7) SLN injury was diagnosed when new hoarseness or decreased pitch was accompanied by laryngoscopic evidence of impaired ipsilateral cricothyroid function 1 week postoperatively.

### Sample size calculation and statistical analysis

The sample size was calculated based on the quality of airway anesthesia grade (on a scale of 0 to 5, see Table 2), using preliminary data obtained from 20 consecutive eligible patients at our center with PASS 19.0 software (PASS, USA). The mean grade of the quality of airway anesthesia was 0.4 and 1, with a common standard deviation of 0.52, 0.94 in the TCP and FIB groups. The calculated sample size was 27 participants per group, with a power of 0.8 and an alpha error of 0.05. The total required recruitment was 60 patients, accounting for a dropout of 10%. Although the priori sample size was calculated using a two-sample *t*-test, we treated the ordinal primary outcome as continuous solely for this estimation, as the pilot sample (*n* = 20) was too small to yield stable rank-based or logistic parameters. Asymptotic relative-efficiency theory indicates that the Mann–Whitney *U* test preserves ≥95% power under this parametric estimate, ensuring that *n* = 60 maintains ≥80% power for the non-parametric analysis actually used. The primary outcome was analyzed using the Mann–Whitney *U* test because of the observed distribution of the data after power analysis.

Data analysis was performed using SPSS version 19.0 (IBM Corp., Armonk, NY, USA). Normally distributed continuous data are presented as mean (standard deviation) and were analyzed using Student’s *t*-test. Non-normally distributed continuous data are presented as median (IQR, interquartile range) and were analyzed using the Mann–Whitney *U* test. Categorical data are presented as numbers or percentages and were analyzed using the chi-square test. *P* values *<*0.05 were considered statistically significant.

## Results

### Anatomical study

**Figure 2.**
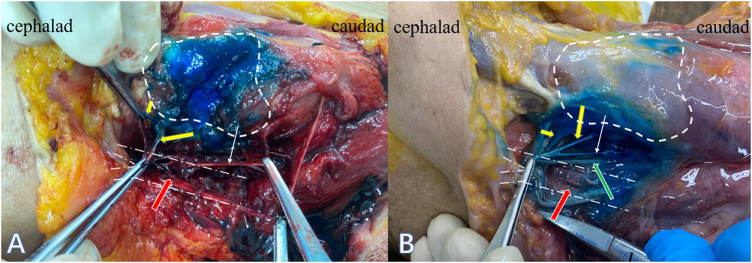
Anatomical dissection. (A) The short yellow arrow indicated superior laryngeal artery, the long yellow arrow indicated internal branch of SLN, the white arrow indicated external branch of SLN. (B) The short yellow arrow indicated superior laryngeal artery, the long yellow arrow indicated internal branch of SLN, the white arrow indicated external branch of SLN, the green arrow indicated the superior thyroid artery, the red arrow indicated the carotid artery, and the dotted line indicated the boundary of thyroid cartilage.

Two cadavers received bilateral TCP block. The spread of the dye was limited to the superior border of hyoid bone cephalad, inferior border of cricoid cartilage caudad, and medial border of internal carotid artery laterally. In all dissections, the thyrohyoid membrane was stained by the dye. The internal branch of the SLN and the superior laryngeal artery were stained in all cases (4/4). However, in one of four sides, the external branch of SLN was not stained. None of the recurrent laryngeal nerves were stained (Fig. 2).

### Clinical study

**Figure 3.**
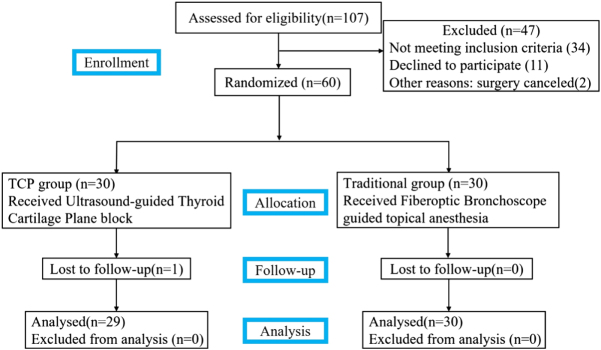
Study flow diagram of patient recruitment.

We randomized sixty consenting patients with an anticipated difficult airway in our trial. One patient in the TCP group transferred to the ICU due to massive intraoperative surgical bleeding that occurred during tumor resection, and thus was excluded from the trial. Finally, 59 patients finished our trial (Fig. 3). The demographic profile and airway parameters were all comparable between the two groups, confirming successful randomization (Fig. 3 and Table 1).

**Table 1:** Baseline demographics, airway parameters, and randomization balance. Values are mean (standard deviation) or number (proportion), apart from age, which is presented as median (IQR).

Index	FIB group (*n* = 30)	TCP group (*n* = 29)	*P*
Age (yr)	57.5 (51.5, 61.3)	59 (52, 62)	0.538
Sex (male/female)	21/9	19/10	0.713
BMI (kg·m^−2^)	21.3 (2.1)	22.4 (3.2)	0.140
ASA (I/II)	13/17	9/20	0.329
Limited cervical spine mobility	22 (73.3)	19 (65.5)	0.514
Limited mouth opening	2 (6.7)	0 (0)	0.492
Full stomach	6 (20)	10 (34.5)	0.211
Mallampati class (1/2/3/4)	13/12/4/1	10/13/5/1	0.913
Mean thyromental distance(cm)	6.8 (0.5)	7.0 (0.3)	0.202

ASA, American Society of Anaesthesiologists; BMI, body mass index.

The quality of airway anesthesia was significantly better in TCP group with lower quality of airway anesthesia grade as compared to FIB group (median [IQR], 0 [0–0] vs 1 [0–2], *P* < 0.001) (Table 2). MAP and heart rate (HR) were more stable in the TCP group than the FIB group once the tracheal tube was inserted into the trachea (*P* < 0.05), as shown in Table 3. There was no significant difference in sore throat and hoarseness between the two groups (Supplemental Digital Content Table 1, available at: http://links.lww.com/JS9/G831). Airway hemorrhage or nerve injury was not observed in either group.

**Table 2:** Comparison of quality of airway anesthesia grade (0 = excellent, 4 = very poor), tube tolerance score, and time needed between two groups. Values are presented as median (IQR) or number (%).

Index	FIB group (*n* = 30)	TCP group (*n* = 29)	Difference (95% CI)	*P*
Quality of airway anesthesia grade	1 (0.2)	0 (0.0)	1 (0.1)	<0.001
0. excellent, no coughing or gagging	11 (36.7)	24 (82.8)	46.1% (24.0%, 68.1%)	<0.001
1. good, mild coughing and/or gagging	11 (36.7)	4 (13.8)	−22.9% (−44.2%, −1.5%)	0.044
2. fair, moderate coughing and/or gagging	7 (23.3)	1 (3.4)	−19.9% (−36.4%, −3.4%)	0.026
3. poor, severe coughing and/or gagging	1 (3.3)	0	−3.0% (−9.8%, 0.3%)	0.321
4. very poor, very severe coughing and/or gagging	0	0	0	1.000
Tube tolerance score after intubation	1 (1.1)	1 (1.1)	0 (0.0)	0.168
cooperation, restlessness	28 (93.3)	29 (100)	6.7% (−2.3%, 15.6%)	0.157
mild resistance	2 (6.7)	0	−6.7% (−15.6%, 2.3%)	0.157
severe resistance	0	0	0	1.000
Performing time (seconds)	–	86.8 (27.3)	–	–

**Table 3:** Data of hemodynamic profile and Ramsay score during procedure. Values are mean (standard deviation) or median (interquartile range).

Index		FIB group (*n* = 30)	TCP group (*n* = 29)	*P*
MAP (mmHg)	T_0_	101 (12.4)	100.5 (15.6)	0.881
	T_1_	99.1 (14.2)	100.5 (16.1)	0.721
	T_2_	109.7 (13.2)	101.7 (16.1)	0.041
	T_3_	94.5 (18.3)	95.0 (16.8)	0.907
HR (beats/min)	T_0_	81.5 (16.9)	81.7 (15.8)	0.971
	T_1_	79.8 (17.8)	78.6 (14.0)	0.773
	T_2_	88.2 (16.6)	80.0 (10.8)	0.030
	T_3_	78.2 (17.7)	73.8 (13.3)	0.286
Ramsay score	T_0_	2 (2.2)	2 (2.2)	1
	T_1_	3 (2.3)	2 (2.3)	0.387
	T_2_	3 (2.3)	2 (2.3)	0.659
	T_3_	3 (2.3)	3 (2.3)	0.676

T_2_, immediately after tracheal tube insertion.

## Discussion

In this study, we demonstrate that ultrasound-guided TCP block provides safe and effective anesthesia for ATI. Our anatomical study showed that the dye spread superficially to the thyrohyoid membrane and effectively stained the internal branch of the SLN in all cadavers, with only one side lacking the external branch of the SLN staining. Our findings suggest that the anatomical location and injection technique used in the TCP block are effective in delivering the local anesthetic to the internal branch of the SLN. In the clinical trial, the TCP group demonstrated superior quality of airway anesthesia compared to the FIB group. TCP block provides better topical anesthesia of the laryngeal structures, with fewer episodes of coughing/gagging and smaller hemodynamic excursions, although absolute changes in HR and MAP remained within normal limits and are unlikely to be clinically important.

Previous clinical practice involved performing bilateral SLN blocks targeting the SLN space using a parasagittal approach^[33]^. We observed that the local anesthetic diffused beneath the thyrohyoid muscle and reached the TCP. This observation suggested that reversing the direction of the injection might be feasible. The essence of our technical modification is to shift the target from the relatively indistinct thyrohyoid membrane in the SLN space to the thyroid cartilage^[27]^. The thyroid cartilage, the largest cartilaginous structure of the larynx, can be easily identified through both palpation and ultrasonography. The bilateral laminae of the thyroid cartilage offer a protective anatomical barrier for the airway, allowing our TCP block procedure to be safely conducted within this protective range. Consequently, our TCP block is anatomically distant from the airway mucosa, nerves, and vessels, thereby potentially reducing the risk of accidental puncture of these structures.

Compared with “spray-as-you-go” topicalization, TCP block reduced the median (IQR) intubation condition grade from 1 (0–2) to 0 (0–0). More importantly, the proportion of patients with optimal conditions (grade 0 or 1) increased from 76% to 99%, yielding a number-needed-to-treat of approximately four. In the context of ATI, grade 2 (moderate cough/gag) frequently requires additional topical lidocaine or sedation and is perceived by operators as “clinically difficult.” Thus, the 19.9% patients shifting from grade 2 to grade 0/1 may represent a meaningful reduction in procedural stress, operator frustration, and cardiovascular stimulation.

To facilitate the SLN block, the SLN or artery^[19,20,24]^, thyrohyoid membrane was attempted to be distinguished under ultrasonography^[19,33–35]^. Yet, the accurate localization of these relatively indistinct landmarks requires abundant clinical experience and high-quality ultrasound images, which may lead to suboptimal block efficacy and inadvertent nerve or vessel injury^[15,26]^. These challenges significantly impede the widespread adoption of the SLN technique for ATI. The thyroid cartilage was used as an alternative sonographic anchor when visualization of the SLN or artery became difficult. Moreover, this modified approach had the potential to minimize direct injection of airway mucosa, artery, or nerve by choosing a route anatomically distant away^[27]^.

Preparation before ATI is often time-consuming, with approximately 3–20 minutes reported, challenging patients’ patience and comfort levels^[6,7,36,37]^. A recent meta-analysis concluded that airway nerve blocks may reduce the intubation time during awake intubation^[38]^. The TCP block eliminates the need for fiberoptic bronchoscope techniques to anesthetize the vocal cords, which may be time-consuming. The procedure time for TCP block is relatively short, with an average block performance time of approximately 86.8 seconds in our study. Although we did not compare the total time needed to accomplish the ATI procedure in our trial, we believe that this novel approach should spare more time and improve patient comfort in clinical settings.

In our trial, we used 2% lidocaine 5 ml to anesthetize the SLN, identical to volumes reported for the conventional SLN block method^[27,34]^. In our previous study, nearly 50% of the patients experienced hoarseness immediately post SLN blockade. We speculated that hoarseness may serve as a simple indicator of a successful SLN block^[39]^. In Ramkumar’s study, all patients receiving an SLN block had hoarseness postoperatively^[12]^. Despite this, hoarseness rates were similar between groups. The external branch of the SLN innervates the cricothyroid muscle and is the principal motor regulator of vocal-cord tension. Inadvertent blockade of the external branch can produce transient hoarseness. Cadaveric data suggest that the external branch is spared in 25% of injections, most likely because the injectate remains medial to the thyroid cartilage lamina and deep to the thyrohyoid muscle, anatomically separated from the more lateral course of the motor branch. This selective sensory predominance is a practical advantage when prolonged phonation impairment is undesirable.

Our study also had several limitations. First, it was conducted at a single center with a modest sample of ASA I–II adults aged 18–65 years and body mass index <30 kg m^−2^; larger, multicenter trials including elderly and obese patients are required before widespread adoption. Second, anatomical validation was performed in only two cadavers; further dissection studies are needed to quantify the consistency of injectate spread across diverse cartilage morphologies. Third, we did not compare TCP block with other ultrasound-guided SLN approaches; head-to-head trials are required to determine relative ease and complication rates. Fourth, all blocks were performed by two experienced anesthesiologists; the learning curve for novices remains undefined. Finally, we did not record the total time required to complete ATI, a metric that could provide additional insight into efficiency.

## Conclusion

In summary, compared with fiberoptic bronchoscope-guided topical anesthesia, ultrasound-guided TCP block improved intubation conditions and hemodynamic stability in patients undergoing ATI. Our anatomical and clinical study results demonstrate that the TCP block is effective and safe for SLN blockade. While further research is needed to optimize its application, the TCP block has the potential to become a valuable addition to the management of difficult airways.

## Data Availability

All data relevant to the study are included in the article. The data supporting the findings of this study are available from the corresponding author upon reasonable request.

## Research registration unique identifying number (UIN)

ClinicalTrials.gov (NCT06065475).

## Assistance with the study

The authors thank Zhiyuan Mao, Lei Li, and Yongjie Zhang, from the Department of Human Anatomy, School of Basic Medical Sciences, Nanjing Medical University, for their help with the anatomical dissection study.
